# Using routine emergency department data for syndromic surveillance of acute respiratory illness, Germany, week 10 2017 until week 10 2021

**DOI:** 10.2807/1560-7917.ES.2022.27.27.2100865

**Published:** 2022-07-07

**Authors:** T. Sonia Boender, Wei Cai, Madlen Schranz, Theresa Kocher, Birte Wagner, Alexander Ullrich, Silke Buda, Rebecca Zöllner, Felix Greiner, Michaela Diercke, Linus Grabenhenrich

**Affiliations:** 1 Robert Koch Institute, Department for Infectious Disease Epidemiology, Berlin, Germany; 2 Charité–Universitätsmedizin Berlin, corporate member of Freie Universität Berlin and Humboldt Universität zu Berlin, Institute of Public Health, Berlin, Germany; 3 Robert Koch Institute, Department for Methodology and Research Infrastructure, Berlin, Germany; 4 Health Protection Authority, Frankfurt am Main, Germany; 5 Department of Trauma Surgery, Otto von Guericke University Magdeburg, Magdeburg, Germany; 6 AKTIN–Emergency Department Data Registry, Magdeburg/Aachen, Germany; 7 Institute for Occupational and Maritime Medicine (ZfAM), University Medical Center Hamburg-Eppendorf (UKE), Hamburg, Germany

**Keywords:** public health surveillance, COVID-19, respiratory tract infections, emergency service, hospital

## Abstract

**Background:**

The COVID-19 pandemic expanded the need for timely information on acute respiratory illness at population level.

**Aim:**

We explored the potential of routine emergency department data for syndromic surveillance of acute respiratory illness in Germany.

**Methods:**

We used routine attendance data from emergency departments, which continuously transferred data between week 10 2017 and 10 2021, with ICD-10 codes available for > 75% of attendances. Case definitions for acute respiratory infection (ARI), severe acute respiratory infection (SARI), influenza-like illness (ILI), respiratory syncytial virus infection (RSV) and COVID-19 were based on a combination of ICD-10 codes, and/or chief complaints, sometimes combined with information on hospitalisation and age.

**Results:**

We included 1,372,958 attendances from eight emergency departments. The number of attendances dropped in March 2020 during the first COVID-19 pandemic wave, increased during summer, and declined again during the resurge of COVID-19 cases in autumn and winter of 2020/21. A pattern of seasonality of respiratory infections could be observed. By using different case definitions (i.e. for ARI, SARI, ILI, RSV) both the annual influenza seasons in the years 2017–2020 and the dynamics of the COVID-19 pandemic in 2020/21 were apparent. The absence of the 2020/21 influenza season was visible, parallel to the resurge of COVID-19 cases. SARI among ARI cases peaked in April–May 2020 (17%) and November 2020–January 2021 (14%).

**Conclusion:**

Syndromic surveillance using routine emergency department data can potentially be used to monitor the trends, timing, duration, magnitude and severity of illness caused by respiratory viruses, including both influenza viruses and SARS-CoV-2.

## Introduction

With the coronavirus disease 2019 (COVID-19) pandemic, having timely information on acute respiratory illness at population level has become increasingly important to support public health action and healthcare planning. To monitor the burden of respiratory illnesses, robust surveillance systems are essential. In Germany, both COVID-19 and the measures to contain the spread of its causative pathogen – severe acute respiratory syndrome coronavirus 2 (SARS-CoV-2), have had an effect not only on the transmission of seasonal influenza viruses, but also other respiratory viruses [1,2]. These changes in the epidemiological situation affect routine surveillance systems monitoring the epidemiology and virology of respiratory illnesses [3]. Therefore, a system for collecting further timely data on these illnesses is helpful to understand their impact on population health and to support healthcare management.

As public health institutes are planning to transition from COVID-19 emergency surveillance to routine surveillance of all respiratory pathogens, syndromic surveillance of acute respiratory illness, i.e. cross-pathogen symptomatic cases, plays a prime role [4]. In Germany, certain syndromic surveillance systems for acute respiratory infection (ARI) had already been implemented before the pandemic: internet-based syndromic monitoring of ARI in the general population (*GrippeWeb*, FluWeb) [5]; sentinel surveillance of ARI consultations in primary care, including virological surveillance (Influenza Working Group [6]) and sentinel surveillance of severe acute respiratory infection (SARI) among hospitalised patients using International Classification of Diseases (ICD)-10 diagnostic codes (ICOSARI) [7]. In addition, defined respiratory diseases, such as COVID-19 and influenza are monitored through mandatory notifications by clinicians and laboratories, within the framework of the Protection against Infection Act.

The availability of electronically-collected routine medical data, such as emergency department data, allows for an extended approach in applied epidemiology and public health surveillance [8]. Syndromic surveillance using routine emergency department data can be a fast approach to report and detect changes in healthcare utilisation, without creating an additional administrative burden. In the United Kingdom (UK) [9], France [10] and other countries, syndromic surveillance systems based on emergency department data have been established [11,12], and have previously provided timely public health insight upon extraordinary events such as extreme weather occurrence [13], mass gatherings (e.g. the Olympic Games in 2012 [14]), or during the COVID-19 pandemic [15].

Previous analyses of emergency department data in Germany have successfully allowed to detect syndromes for unspecific gastrointestinal infections, allowing their surveillance, including that of their seasonal fluctuation [16]. Ways to describe ARI cases in order to monitor them have also been developed [17]. Since the end of June 2020, the weekly Emergency Department Situation Reports have presented data from the routine documentation of up to 21 selected emergency departments in the country [18]. These reports showed that parallel to the COVID-19 pandemic and the associated control measures starting mid-March 2020, emergency-department attendances dropped [19].

Nationwide public health and social measures directed at the COVID-19 pandemic commenced in February–March 2020 [20-22]. The measures comprised steps to increase physical distancing, such as a ‘contact ban’ allowing only a maximum of two people from different households to meet in person (with minimum 1.5 metres distancing), and a closure of most public spaces including schools, non-essential shops and many businesses [20-22]. By April–May 2020, public health and social measures were gradually eased. Nevertheless, large-scale testing focusing on symptomatic suspected COVID-19 cases, contact of cases and people in vulnerable settings stayed in place. Face-mask use was required in certain settings (e.g. public transport, healthcare facilities, shops) and physical distancing also continued to be strongly recommended. People traveling to Germany from areas classified as high-risk for COVID-19 had to undergo a 14-day quarantine. During the summer months of 2020, emergency department attendances partially increased again [19]. With the resurge of COVID-19 cases in the autumn of 2020, many public health and social measures were re-implemented, such as a working from home policy, the mandatory reduction of contacts and the closure of most public spaces (including non-essential shops and some hospitality-industry businesses serving food and drinks – e.g. bars and restaurants) [21]. By December 2020, schools were also closed. During the autumn and winter of 2020/21, coincidental to the resurge of COVID-19 cases and implementation of measures, the number of emergency-department attendances decreased again to about one quarter below the 2019 average. From March 2021, gradual lifting of measures started, followed by introducing a ‘3G’ policy (i.e. requiring proof of either a negative COVID-19 lateral flow test (*‘Getestet’*), completed course of vaccination (*‘Geimpft’*), or recent recovery (*’Genesen’*)) when visiting shops, some hospitality-industry businesses and when practicing sports [21]. Emergency attendances remained below the 2019 average until the time of analysis and writing (April 2021) [18].

In this work, we aimed to describe emergency-department attendances for acute respiratory illness in Germany over time, for the purpose of developing and implementing syndromic surveillance. The aim was also to gain more insight into the epidemiology of this type of illness and to obtain a greater understanding of how related presentations to emergency care affect attendance levels. Furthermore, we explored the impact of the COVID-19 pandemic, and its associated public health and social measures, on emergency-care seeking behaviour concerning this type of illness.

## Methods

### Study design

We conducted a retrospective observational study to explore the potential of routine emergency department data for syndromic surveillance of acute respiratory illness in Germany. Following a process of data curation, quality management and exploration, and defining syndromic case definitions, we described emergency department attendances over time for the period from 6 March 2017 up to and including 13 March 2021.

### Setting

Data used in this analysis were provided through two networks of German emergency departments which continuously transfer anonymised routine health data to the Robert Koch Institute (RKI, Germany’s national public health institute) [23]: the AKTIN – Emergency Department Data Registry [24] and the ‘Erkennung und Steuerung Epidemischer Gefahrenlagen’ (ESEG)-project – detection and control of epidemic situations (http://www.rki.de/eseg). Germany has over 1,000 emergency departments that differ in size and level of care (basic/extended/comprehensive emergency care) [25]. There are no barriers to accessibility, as patients can be admitted by ambulance, referred by a general practitioner or even arrive by their own means (i.e. without a referral) as ‘walk-in’ patients. Emergency departments participate and share data on a voluntary basis. At the RKI, these routine health data from the different sources are merged and processed using the NotaufnahmeKernDatensat*z* (NoKeDa) data model [26], when the date of visit, age, and sex information are available. Observations are recorded by visit (attendance) and not by person; multiple visits by the same person cannot be linked.

### Study population

Emergency attendance data were exported from the RKI server on 13 April 2021, using SUMO-DB Release 3.0.3 (database population). In addition to the data release, data quality summary reports were generated, including information on completeness of attendances and variables. The data availability and quality could be assessed, based visual aids/graphs and on descriptive statistics. For the purpose of this work, a subset of emergency departments was included (study population): departments, which continuously transferred data on at least one attendance per day for the period from 6 March 2017 (week 10 2017) up to and including 13 March 2021 (week 10 2021), with ICD-10 diagnostic codes for > 75% of the attendances.

### Variables

The following variables were included for analysis: emergency department/hospital, age (collected in age groups: 0–2 years, 3–4 years, followed by 5-year groups, and ≥ 80 years), sex (male/female/other), triage (codes 1 to 5, higher code meaning less urgent), diagnosis (ICD-10 codes, when labelled confirmed/suspected), chief complaints (according to the Canadian Emergency Department Information System–Presenting Complaint List (CEDIS-PCL) [27] or Manchester Triage System (MTS) [28]), referral, mode of transport, and disposition (e.g. hospital admission). ‘Potential missing data’ refers to the absence of documentation; all data are based on routine emergency care at the respective sites, based on routine documentation practices.

### Case definitions

We used a set of case definitions in agreement with the established syndromic surveillance systems of acute respiratory illness at RKI and based on (inter)national standards [29,30]. Case definitions were based on a combination of ICD-10 diagnostic codes, or chief complaints, sometimes combined with disposition and age. Case criteria and case classifications (i.e. probable, confirmed) for ARI, SARI, influenza-like illness (ILI), respiratory syncytial virus infection (RSV) and COVID-19 are summarised in Supplementary Table 1. Combined case definitions were used for the primary analysis; i.e. cases of ILI, RSV and COVID-19 were either probable and/or confirmed.

### Statistical analysis

Attendances and cases were analysed as aggregated counts by week. Weekly numbers of all cases that met the case definitions (see above and Supplementary Table 1) were plotted as absolute counts, as well as number of cases per 1,000 attendances to adjust for fluctuations in attendances (i.e. a change in the denominator) over time. Cases of ARI and SARI were explored as individual indicators, as well as through calculating the percentage of severe cases among ARI cases (SARI/ARI). Probable and confirmed cases of ILI, RSV and COVID-19 were explored as separate and combined overall case numbers (probable + confirmed cases).

The number of weekly attendances were described and summarised for the period before the pandemic (i.e. before 2 March in week 10 2020) and during the COVID-19 pandemic. The different phases of the COVID-19 pandemic in Germany were categorised following Schilling et. al [31]: (i) pre-pandemic and sporadic cases up to and including week 09 2020, (ii) first COVID-19 wave in weeks 10 2020–20 2020; (iii) summer plateau in weeks 21 2020–39 2020; (iv) second COVID-19 wave in weeks 40 2020–10 2021. Because the number of weekly attendances were not normally distributed, Wilcoxon rank sum test was used for simple comparisons of the distribution of weekly cases per 1,000 attendances before and during the pandemic. Next, time series were decomposed to visually investigate the seasonality and trend over time; a decomposition of the COVID-19 time series of 1 year was not possible because the series was too short (less than two periods).

Data were analysed using R statistical software, using the *tidyverse, xts* and *zoo* packages [32]. The aggregated data of the attendances and cases (ARI, SARI, ILI, RSV, COVID-19) by week used for the analysis, are provided in the Supplemental Material.

## Results

The study population included 1,372,958 attendances between 6 March 2017 (week 10 2017) up to and including 13 March 2021 (week 10 2021) from eight emergency departments. The included departments provided comprehensive (5 departments) and extended (3 departments) emergency care in seven federal states of Germany (Baden-Württemberg, Bavaria, Berlin, Brandenburg, Hesse, Lower Saxony, Saxony).

The general characteristics of the emergency department attendances are summarised in the Table. The overall sex (48% female) and age (6% ≤ 2 years, 8% 3–9 years; 8% 10–19 years; 17% 20–39 years; 25% 40–59 years; 21% 60–79 years; 15% ≥ 80 years) distributions remained similar over the full observation period, but some drops in emergency attendances were however apparent during the first and second pandemic waves. During these times, attendances in the 0–19-year age group seemed to undergo the largest decrease (Supplementary Table 2 and Supplementary Figure 1).

**Table:** Summary of total emergency department attendances, between week 10 2017 up to and including week 10 2021, for the full observation period, and stratified by time before and during the COVID-19 pandemic, Germany, 6 March 2017–13 March 2021 (n = 1,372,958)

Attendances	Period (weeks)				Total n = 1,372,958	
	10 2017–09 2020 n = 1,082,850		10 2020–10 2021 n = 290,108			
	Number	%	Number	%	Number	%
**Age (in years)**						
**0–2**	56,356	5.2	18,682	6.4	75,038	5.5
**3–4**	40,817	3.8	7,209	2.5	48,026	3.5
**5–9**	56,330	5.2	11,479	4.0	67,809	4.9
**10–14**	43,080	4.0	9,383	3.2	52,463	3.8
**15–19**	49,574	4.6	11,580	4.0	61,154	4.5
**20–39**	183,765	17.0	47,380	16.3	231,145	16.8
**40–59**	269,240	24.9	75,096	25.9	344,336	25.1
**60–79**	227,639	21.0	65,311	22.5	292,950	21.3
**≥ 80**	156,049	14.4	43,988	15.2	200,037	14.6
**Sex**						
**Female**	524,790	48.5	140,161	48.3	664,951	48.4
**Male**	557,971	51.5	149,937	51.7	707,908	51.6
**Other**	89	0.0	10	0.0	99	0.0
**Triage**						
**Immediate**	11,494	1.1	3,795	1.3	15,289	1.1
**Very urgent**	140,416	13.0	42,924	14.8	183,340	13.4
**Urgent**	394,089	36.4	116,371	40.1	510,460	37.2
**Standard**	442,466	40.9	102,638	35.4	545,104	39.7
**Not urgent**	47,274	4.4	13,041	4.5	60,315	4.4
**Missing**	47,111	4.4	11,339	3.9	58,450	4.3
**Transport**						
**Patient transport**	25,134	2.3	6,289	2.2	31,423	2.3
**Ambulance**	102,827	9.5	42,768	14.7	145,595	10.6
**Emergency ambulance**	55,512	5.1	16,889	5.8	72,401	5.3
**Helicopter**	30,092	2.8	274	0.1	30,366	2.2
**Other**	170,045	15.7	65,628	22.6	235,673	17.2
**Missing**	699,240	64.6	158,260	54.6	857,500	62.5
**Referral**						
**Hospital/transfer**	15,561	1.4	5,022	1.7	20,583	1.5
**Ambulatory emergency service outside hospital**	16,636	1.5	4,294	1.5	20,930	1.5
**Ambulatory emergency practice within hospital**	10,196	0.9	2,354	0.8	12,550	0.9
**Not referred by physician**	258,764	23.9	89,385	30.8	348,149	25.4
**Other**	133,834	12.4	17,805	6.1	151,639	11.0
**Emergency service**	191,286	17.7	65,478	22.6	256,764	18.7
**Medical practitioner**	54,711	5.1	16,092	5.5	70,803	5.2
**Missing**	401,862	37.1	89,678	30.9	491,540	35.8
**Disposition**						
**Death**	5,788	0.5	109	0.0	5,897	0.4
**Discharge against medical advice**	6,611	0.6	2,170	0.7	8,781	0.6
**Treatment discontinued by patient**	2,406	0.2	589	0.2	2,995	0.2
**Discharge home**	119,779	11.1	42,374	14.6	162,153	11.8
**Release for further treatment by a physician**	15,343	1.4	4,480	1.5	19,823	1.4
**No physician contact**	1,774	0.2	212	0.1	1,986	0.1
**Inpatient admission - operational unit**	3,577	0.3	1,482	0.5	5,059	0.4
**Inpatient admission - monitoring unit**	18,495	1.7	7,322	2.5	25,817	1.9
**Inpatient admission - regular ward**	73,260	6.8	26,125	9.0	99,385	7.2
**External transfer**	31,770	2.9	820	0.3	32,590	2.4
**Other**	42,173	3.9	75	0.0	42,248	3.1
**Missing**	761,874	70.4	204,350	70.5	966,224	70.4

**Figure 1:**
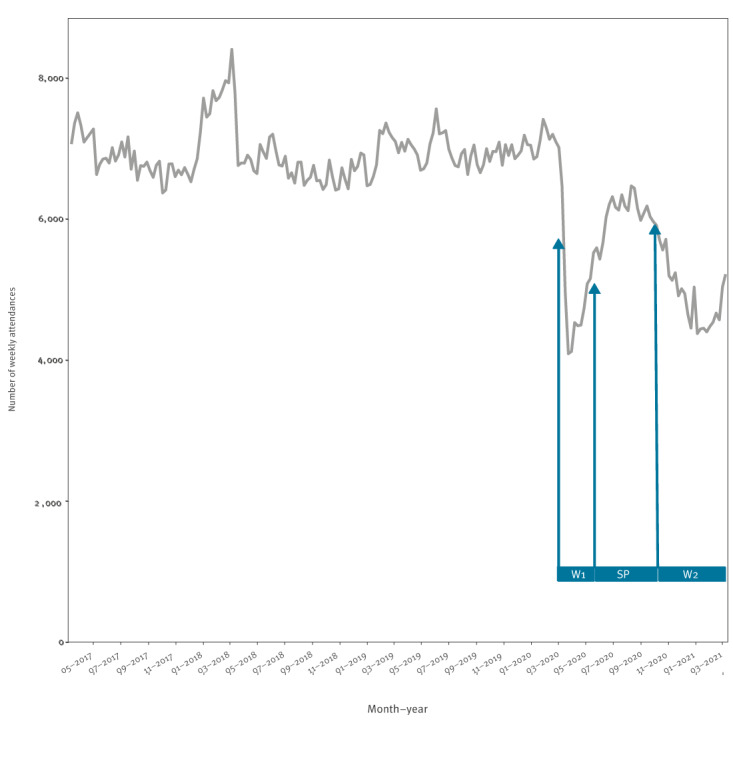
Weekly counts of emergency department attendances, between week 10 2017 and up to and including week 10 2021, Germany, 6 March 2017–13 March 2021 (n = 1,372,958)

W1: first wave of coronavirus disease pandemic; SP: summer plateau; W2: second wave of coronavirus pandemic.

Over time, the absolute number of weekly emergency department attendances dropped during the first and second wave of the COVID-19 pandemic in Germany (Figure 1). The median number of attendances per week dropped from 6,886 (interquartile range (IQR): 6,733–7,104) prior to the pandemic (before week 10 2020) to 4,741 (IQR: 4,498–5,124; p < 0.001) during the first COVID-19 wave (weeks 10 2020–20 2020). Following a recovery phase approaching pre-pandemic attendance rates (median: 6,133; IQR: 6,010–6,202; p < 0.001) during the summer plateau (week 21 2020–39 2020), the weekly attendances declined again to 4,950 (IQR: 4,512–5,212; p < 0.001) during the second COVID-19 wave, which occurred in autumn and winter of 2020/21 (week 40 2020–10 2021).

The weekly attendances differed when comparing the respective phases of the pandemic to the pre-pandemic period, as summarised in Supplementary Figure 2. The highest number of 8,412 attendances per week was counted in week 09 2018, and the lowest number of 4,096 attendances per week was counted in week 12 2020.

### Cases of ARI, SARI, ILI, RSV and COVID-19 over time

**Figure 2:**
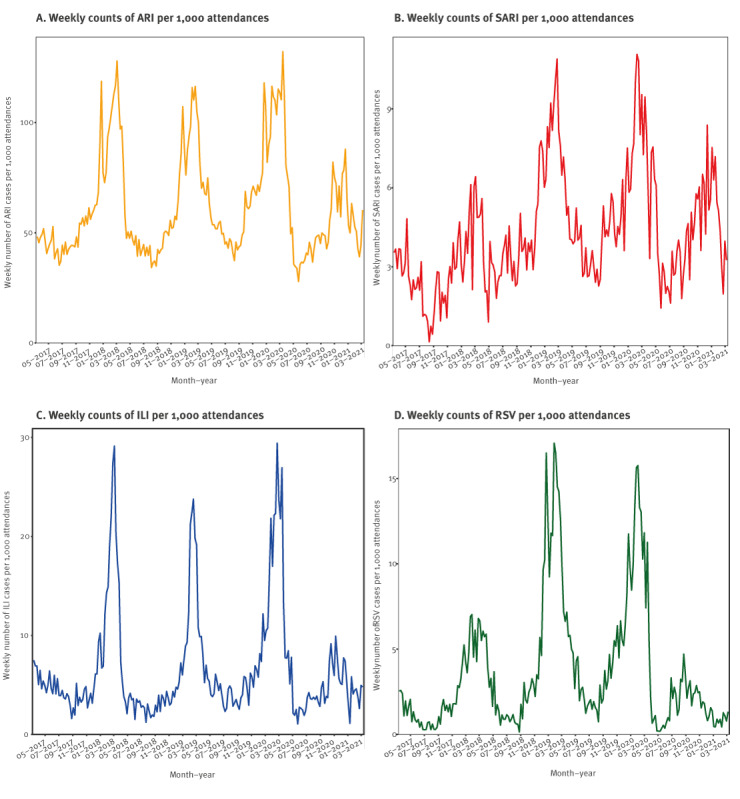
Weekly counts of cases of (A) ARI, (B) SARI, (C) ILI, (D) RSV cases per 1,000 emergency department attendances, Germany, 6 March 2017–13 March 2021

**Figure 3:**
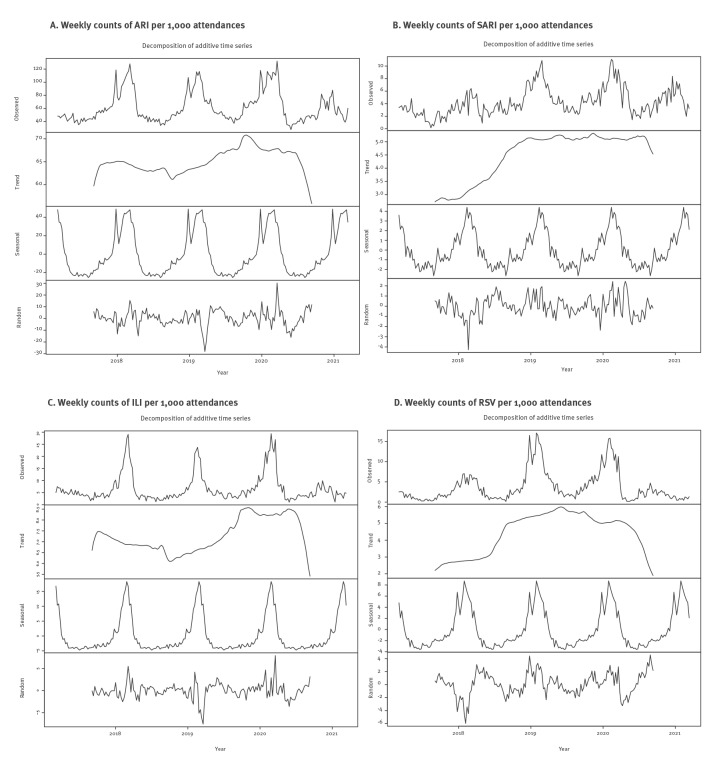
Decomposed time series of the weekly counts of cases of (A) ARI, (B) SARI, (C) ILI and (D) RSV per 1,000 emergency department attendances, Germany, 6 March 2017–13 March 2021

During the full, 4-year observation period, seasonal patterns were visible for weekly ARI, SARI, as well as probable/confirmed ILI and RSV cases per 1,000 attendees (Figure 2). These patterns were also observed per absolute weekly case counts (Supplementary Figure 3). The decomposed time series analysis suggested seasonal factors of ARI, SARI, ILI, and RSV cases (Figure 3).

ARI: acute respiratory infection; SARI: severe acute respiratory infection; ILI: influenza-like illness; RSV: respiratory syncytial virus infection.

Cases of ILI and RSV both include probable and/or confirmed cases. Only one case definition was respectively used for ARI and SARI.

ARI: acute respiratory infection; SARI: severe acute respiratory infection; ILI: influenza-like illness; RSV: respiratory syncytial virus infection.

Cases of ILI and RSV both include probable and/or confirmed cases. Only one case definition was respectively used for ARI and SARI.

**Figure 4:**
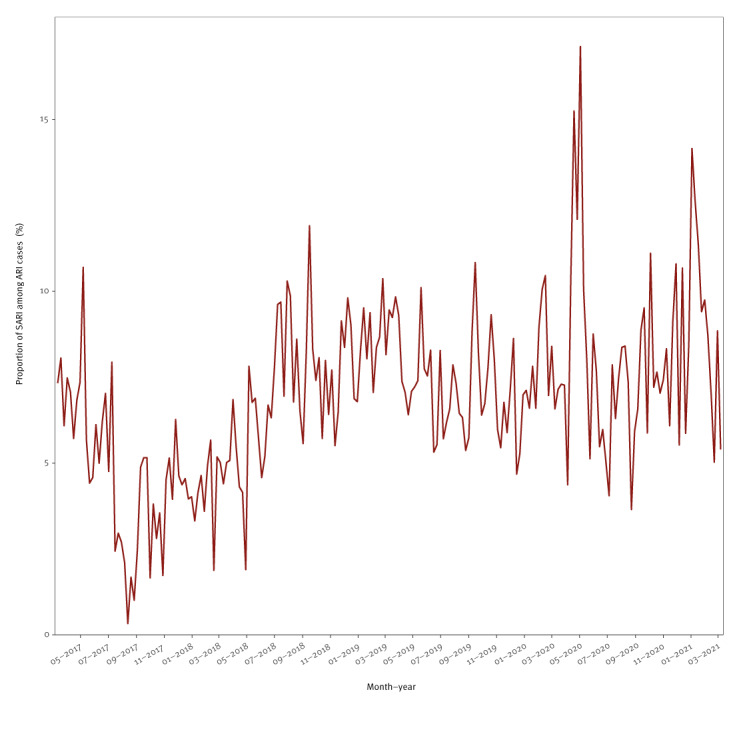
Proportion of severe cases among acute respiratory infection cases, Germany, 6 March 2017–13 March 2021

ARI: acute respiratory infection; SARI: severe ARI.

In the winter of 2020/21, both the absolute and relative ARI case counts were lower than in previous years and showed a downward trend (Figure 2a and 3a). Conversely, the trend of SARI cases remained fairly stable over time, with a small downward turn in the final months of winter (Figure 2b and 3b). This pattern was also visible when assessing the percentage of severe (i.e. SARI) cases among ARI cases (Figure 4). The percentage of SARI cases among ARI cases fluctuated around a median of 6.9% (IQR: 5.3–8.3), was lower in the year 2017 (median: 4.7%; IQR: 3.4–6.2), fairly stable in the years 2018 (median: 6.6%; IQR: 5.1–8.0) and 2019 (median: 7.4%; IQR: 6.4–8.4). The percentage of SARI cases among ARI cases occasionally exceeded 10%, but this excess was only observed in single weeks during pre-pandemic times: week 18 2017, week 30 2018, week 37 2018, week 8 2019, week 20 2019, and week 37 2019. However, during the pandemic, the percentage of SARI cases among ARI cases exceeded 10% twice for ≥ 5 subsequent weeks in a row (week 15 2020 to 19 2020 with a peak at week 18 2020 (17.1%)) and from week 48 2020 to week 2 2021 with a peak at week 53 2020 (14.2%), as displayed in Figure 4.

**Figure 5:**
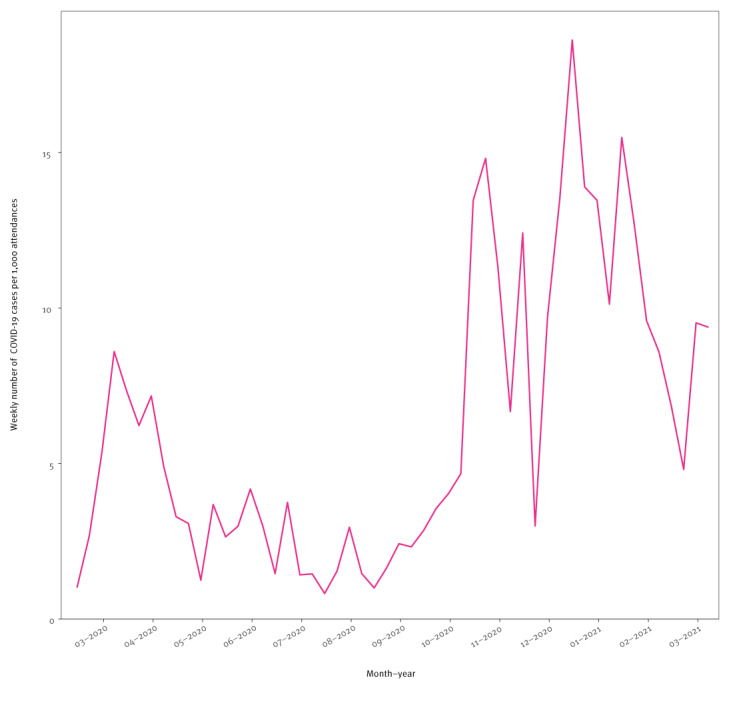
Weekly counts of COVID-19 cases per 1,000 emergency department attendances, Germany, 6 March 2020–13 March 2021

After March 2020, the expected seasonal increases in ILI cases and RSV cases were reduced (Figure 2c-d and 3c-d). In parallel, COVID-19 cases were recorded from March 2020 onwards (Figure 5). Visual inspection of COVID-19 cases showed two periods of increased case numbers, despite the drop of overall attendances, one during April–May of 2020, coinciding with the first pandemic peak, and another with a resurge of cases starting in October 2020 during the second pandemic wave; the number of cases decreased again at the end of the observation period (March 2021). Overall, there were 17% SARI cases among COVID-19 cases (all COVID-19 cases were by definition ARI cases). Of note, weekly ILI, RSV and COVID-19 cases are plotted by probable and possible case classifications, as well as the combined case definition, in Supplementary Figure 4.

COVID-19: coronavirus disease.

Cases of COVID-19 include probable and/or confirmed cases.

## Discussion

This descriptive study provides proof of principle for syndromic surveillance of acute respiratory illness, using routine emergency department records in Germany [33]. Based on data from a selection of eight voluntarily participating departments, a clear pattern of respiratory-infection seasonality (i.e. ARI, SARI, ILI and RSV) could be observed. In addition, both the annual influenza seasons in the 2017/18–2019/20 period and the dynamics of the COVID-19 pandemic in 2020/21 were apparent. Consistent with results from other syndromic surveillance systems, the 2017/18 influenza season seemed more severe than the 2018/19 season, with a higher peak of ARI attendances [31,34,35]. Of note, the abrupt and early end of the 2019/20 influenza wave could be visually identified, as observed by sentinel surveillance in Germany [36], Denmark, Norway and Sweden [37]. The striking absence of an influenza season in 2020/21 [1,38] was also noticeable, parallel to the resurge of COVID-19 cases during the 2020/21 autumn and winter. In 2020 and the beginning of 2021, the percentage of SARI cases among ARI cases peaked twice for ≥ 5 subsequent weeks from April to May 2020 and November 2020 to January 2021, coinciding with the first wave and resurge of COVID-19 cases in Germany [39]. The reduction in ILI and RSV cases during the pandemic period could, at least in part, be explained by the influence of public health and social measures on overall disease transmission, as observed by other surveillance systems in Germany [1,2], and internationally [40].

Overall emergency department attendances dropped substantially following the start of the COVID-19 pandemic in March 2020, increased during summer, and declined again during the re-emergence of the pandemic in the autumn and winter of 2020/21. The shift in attendances required a baseline adjustment for case reporting (change in denominator), by standardising the case count per 1,000 attendances. This reduction in attendances could have been partially due to the advisories to reduce physical contact with healthcare services, and to use telephone services instead (at least before visiting), when possible. This observation fits the overall reduction in hospitalisation as reported by other German hospitals [41,42]. In addition, a reluctance to visit healthcare facilities due to a fear to be exposed to SARS-CoV-2 [43], could also be a reason for a reduction in emergency department attendances. Similar trends were observed in emergency departments in other countries, including the UK [44] and United States [45].

The findings of this study should be interpreted taking the following limitations into account. First, the selected data are from a convenience sample. They are not representative of emergency departments in Germany as a whole. Moreover, as the current study’s aim was to mainly explore the feasibility of using routine emergency-department data for surveillance, differences within and between departments were minimally considered in the data analysis and interpretation. Second, structural changes at the departments during the investigation period, including active interreference of flows of patients with respiratory complaints, could have affected reported attendances and case counts. To fully understand trends in the data, whether affected by a change in infection dynamics, healthcare seeking behaviour, the COVID-19 pandemic and public health and social measures, or structural changes at emergency departments, more information is needed. Seeking this, is, however, beyond the scope of this work. While enabling to timely gather data without creating an additional burden on healthcare workers is a strength of the passive monitoring reported here, a third limitation is that case definitions for the purpose of triage and clinical documentation in the emergency department were not designed for surveillance. Their syndromic nature and heavy reliance on clinical presentation means that laboratory diagnosis is frequently not available or not foreseen, creating potential uncertainties in data interpretation. Shortness of breath, for example, may be a symptom resulting from cardiovascular disease rather than infection by a respiratory pathogen [46]. Furthermore, in this study, information on hospitalisation (disposition), a key variable for SARI cases, was not always available, leading to potential under-reporting of SARI cases. In addition, ICD-10 codes for COVID-19 diagnosis were not available from the beginning of the pandemic, leading to under-reporting of the true number of COVID-19 cases.

Since November 2021, the syndromic surveillance indicators based on the ARI, SARI and confirmed-ILI case definitions in this paper are included in the weekly reporting of the Emergency Department Surveillance in Germany, by the RKI [33]. This updated version of the report includes a data quality table of each variable of the current week. Moving forward, the current ongoing systematic collection, analysis, interpretation, and dissemination of emergency department data for use in public health action, needs to be evaluated, to make targeted recommendations to improve quality, efficiency, and usefulness of the system [47]. Especially in the context of the COVID-19 pandemic, targeted analyses looking into the potential shifts in attendances during different phases of the pandemic are ongoing. Triangulation of data sources and formal comparison with established surveillance systems, including COVID-19 case notifications [48], and other syndromic and virological surveillance systems for acute respiratory illness [5,7], will help to address under-ascertainment, to understand changes in consultation behaviour during public health emergencies and improve interpretation of the data.

COVID-19 poses a challenge for syndromic surveillance with an additional illness among those to monitor. Syndromic surveillance in emergency departments can help provide an overview of healthcare utilisation, independent of the pathogen, for overall public health and healthcare surveillance [49]. Moving forward, increased COVID-19 vaccination roll-out and subsequent adaptation/de-escalation of public health and social measures against SARS-CoV-2 may impact on circulation of other respiratory viruses as well. The almost real-time syndromic surveillance reported here may timely inform public health decision-making.

## Conclusions

The current analysis of emergency department data collected during 4 years identified syndromic cases of (severe) ARI, and could differentiate between ILI, RSV and COVID-19 cases. Clear patterns of seasonality and tendencies over time were identified, for all case definitions, in line with findings reported from established ARI, SARI, ILI and virological surveillance systems [50]. Syndromic surveillance of ARI and SARI in the emergency department could contribute to the overall burden estimates of acute respiratory illness in the upper and lower respiratory tract, which is essential in the transition transfer from COVID-19 emergency surveillance to routine surveillance of all respiratory pathogens [4]. Syndromic surveillance using routine emergency department data has the potential to complement established indicator based, syndromic and virological surveillance systems used to monitor the timing, duration, magnitude and severity of epidemics caused by respiratory viruses, including RSV, influenza virus and SARS-CoV-2. Findings of this study facilitated the implementation of surveillance indicators of ARI, SARI and ILI in the weekly emergency department surveillance reports [33], and cross-pathogen syndromic surveillance of symptomatic cases of respiratory illness in Germany overall [4].

## Supplementary Data

335000 Supplementary Material
