# Supplementary material for: Using routine emergency department data for syndromic surveillance of acute respiratory illness, Germany, week 10 2017 until week 10 2021
Source: Euro Surveill. 2022 Jul 7;27(27):2100865. doi: 10.2807/1560-7917.ES.2022.27.27.2100865 (PMC9264729; doi:10.2807/1560-7917.ES.2022.27.27.2100865)
Supplement: Supplementary Material [file 2100865_SupplementaryMaterial.pdf]

Supplementary Material

This supplementary material is hosted by Eurosurveillance as supporting information alongside the article "Using routine emergency department data for syndromic surveillance of acute respiratory illness, Germany, week 10 2017 until week 10 2021", on behalf of the authors, who remain responsible for the accuracy and appropriateness of the content. The same standards for ethics, copyright, attributions and permissions as for the article apply. Supplements are not edited by Eurosurveillance and the journal is not responsible for the maintenance of any links or email addresses provided therein.

Appendices

Supplemental Table 1. Case definitions, and case classification (if applicable), used for syndromic surveillance based on routine emergency department data.

Case	Definition
Acute respiratory infection (ARI)	- ICD-10 diagnostic code (any): J00-J22, J44.0, B34.9, U07.1, U07.2<br>**OR**<br>- Chief complaint(s):<br>o [MTS: breathing problems **AND** (elevated temperature/fever **OR** suspected sepsis **OR** productive cough **OR** shortness of breath **OR** elevated breathing)]<br>**OR**<br>o [MTS: sore throat **AND** (elevated temperature/fever **OR** suspected sepsis **OR** special risk of infection **OR** known or suspected immunosuppression **OR** travel i.e. report on recent stay abroad **OR** special risk of infection **OR** airway at risk **OR** short of breathing **OR** light/medium/strong pain **OR** unresponsive child **OR** rapid onset **OR** shock **OR** hypersalivation **OR** stridor **OR** drowsy i.e. altered level of consciousness)]<br>**OR**<br>o [MTS: asthma **AND** (elevated temperature/fever **OR** suspected sepsis)]<br>**OR**<br>o [MTS: feeling unwell **AND i**nsufficient breathing]<br>**OR**<br>o [CEDIS-PCL (any): 661, 154, 653, 651, 103, 104]
Severe acute respiratory infection (SARI)	- ICD-10 diagnostic code (any): J09-J22, U07.1, U07.2<br>**AND**<br>- Disposition:<br>o [Inpatient admission: internal transfer, operational unit, monitoring unit, regular ward<br>**OR**<br>o External transfer]

Supplemental Table 1. continued.

Influenza-like illness (ILI)	PROBABLE CASE<br>- ICD-10 diagnostic code (any): J06.-, J12.8, J12.9, J18.-, J22<br>**<u>OR</u>**<br>- Chief complaint(s):<br>o [MTS: breathing problems<br>**<u>AND</u>**<br>(elevated temperature/fever **OR** suspected sepsis **OR** productive cough **OR** shortness of breath **OR** elevated breathing)]<br>**<u>OR</u>**<br>o [MTS: sore throat<br>**<u>AND</u>**<br>(elevated temperature/fever **OR** suspected sepsis **OR** special risk of infection **OR** known or suspected immunosuppression **OR** travel i.e. report on recent stay abroad **OR** special risk of infection **OR** airway at risk **OR** short of breathing **OR** light/medium/strong pain **OR** unresponsive child **OR** rapid onset **OR** shock **OR** hypersalivation **OR** stridor **OR** drowsy i.e. altered level of consciousness)]<br>**<u>OR</u>**<br>o [MTS: asthma<br>**AND**<br>(elevated temperature/fever **OR** suspected sepsis)]<br>**<u>OR</u>**<br>o [MTS: feeling unwell **AND** insufficient breathing]<br>**<u>OR</u>**<br>o [CEDIS-PCL (any): 661, 154, 653, 651, 103, 104]<br>**<u>AND</u>**<br>- Fever:<br>o Temperature ≥38 °C<br>**<u>OR</u>**<br>o Chief complaint:<br>▪ MTS: fever<br>**OR**<br>▪ CEDIS-PCL: 852<br>**<u>OR</u>**<br>o [ICD-10 diagnostic code (any): R50.8, R50.9]<br><br>CONFIRMED CASE<br>- ICD-10 diagnostic code (any): J09, J10.-, J11.-
Respiratory syncytial virus disease (RSV) –	PROBABLE CASE<br>- ICD-10 diagnostic code (any): J12.8, J12.9, J18.-, J20.8, J20.9, J21.8, J21.9, J22<br>**<u>AND</u>**<br>- Age ≤2 years<br><br>CONFIRMED CASE<br>- ICD-10 diagnostic code (any): J12.1, J20.5, J21.0, B97.4
Coronavirus disease 2019 (COVID-19)	PROBABLE CASE<br>- ICD-10 diagnostic code (any): U07.1, U07.2<br><br>CONFIRMED CASE<br>- ICD-10 diagnostic code: U07.1

Supplemental Table 2. Age and sex of emergency attendees over time, broken down by subsequent pandemic phases.

	≤W09 2020 (N=1082850)	W10 2020-W20 2020 (N=55212)	W21 2020-W30 2020 (N=59462)	W31 2020-W39 2020 (N=55697)	≥W40 2020 (N=119737)	Overall (N=1372958)
**Age (years)**						
0-2	56356 (5.2%)	3822 (6.9%)	3767 (6.3%)	3482 (6.3%)	7611 (6.4%)	75038 (5.5%)
3-4	40817 (3.8%)	1591 (2.9%)	1483 (2.5%)	1428 (2.6%)	2707 (2.3%)	48026 (3.5%)
5-9	56330 (5.2%)	2239 (4.1%)	2654 (4.5%)	2467 (4.4%)	4119 (3.4%)	67809 (4.9%)
10-14	43080 (4.0%)	1662 (3.0%)	1979 (3.3%)	2072 (3.7%)	3670 (3.1%)	52463 (3.8%)
15-19	49574 (4.6%)	2073 (3.8%)	2301 (3.9%)	2516 (4.5%)	4690 (3.9%)	61154 (4.5%)
20-39	183765 (17.0%)	9008 (16.3%)	9912 (16.7%)	9392 (16.9%)	19068 (15.9%)	231145 (16.8%)
40-59	269240 (24.9%)	14393 (26.1%)	15604 (26.2%)	14160 (25.4%)	30939 (25.8%)	344336 (25.1%)
60-79	227639 (21.0%)	12393 (22.4%)	13276 (22.3%)	11984 (21.5%)	27658 (23.1%)	292950 (21.3%)
80+	156049 (14.4%)	8031 (14.5%)	8486 (14.3%)	8196 (14.7%)	19275 (16.1%)	200037 (14.6%)
**Sex**						
female	524790 (48.5%)	26482 (48.0%)	28608 (48.1%)	26792 (48.1%)	58279 (48.7%)	664951 (48.4%)
male	557971 (51.5%)	28728 (52.0%)	30851 (51.9%)	28902 (51.9%)	61456 (51.3%)	707908 (51.6%)
other	89 (0.0%)	2 (0.0%)	3 (0.0%)	3 (0.0%)	2 (0.0%)	99 (0.0%)

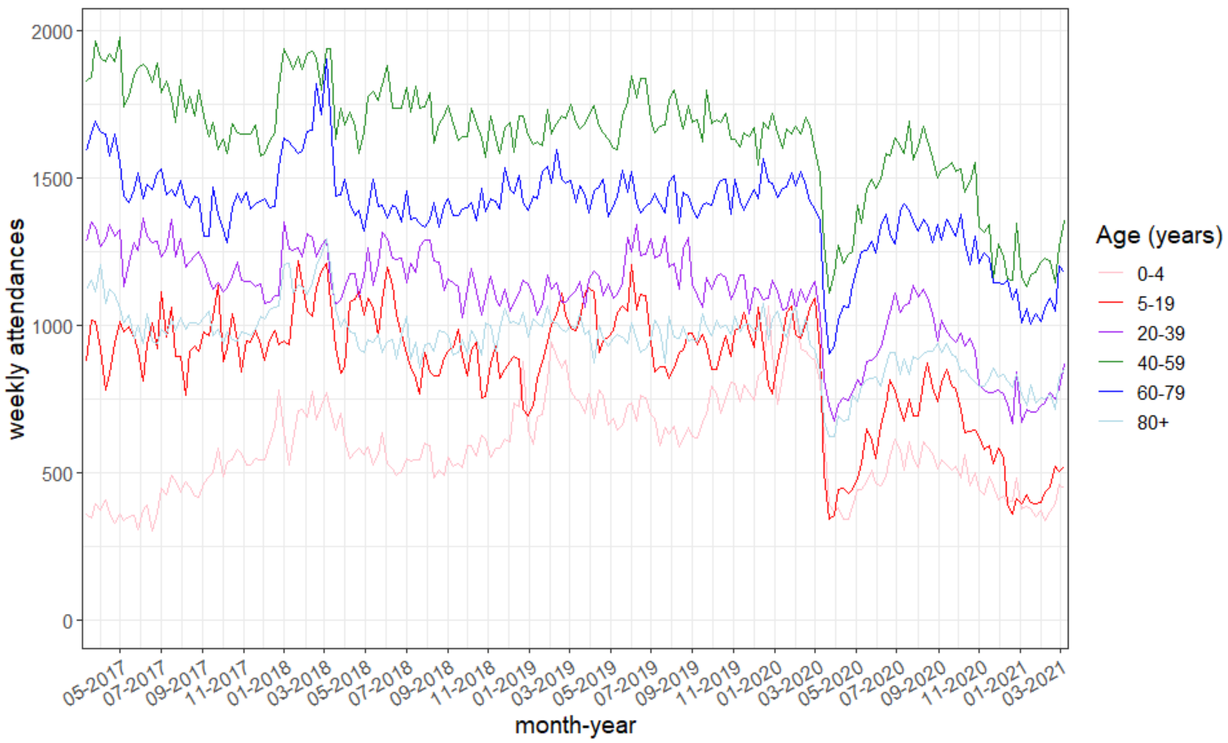

Supplemental Figure 1. Weekly emergency department attendances, by age group.

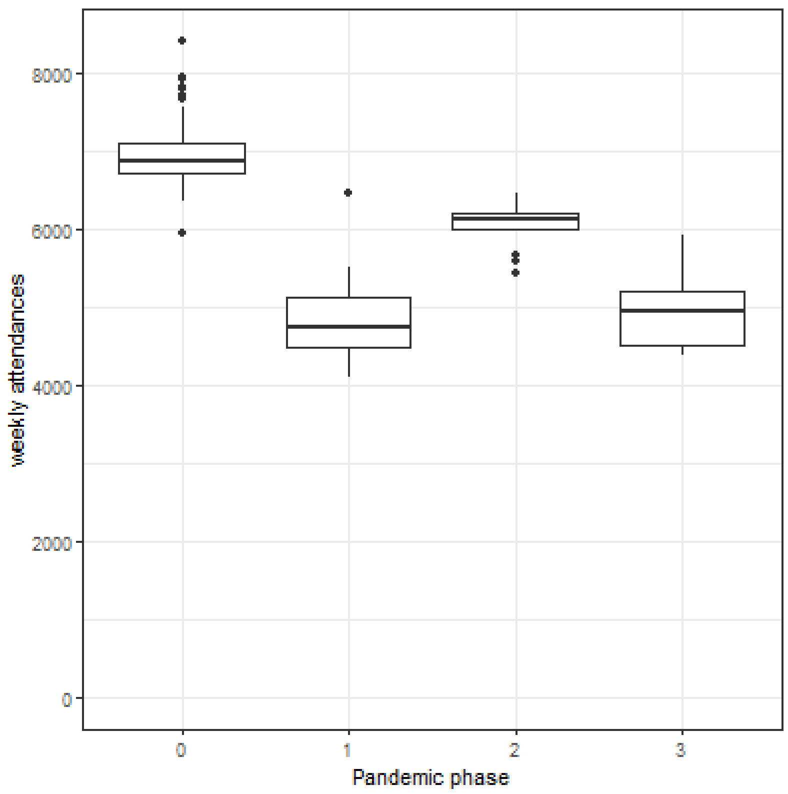

Supplemental Figure 2. Weekly emergency department attendances, a) before and during the pandemic, broken down by subsequent pandemic phases.
All comparisons: Wilcoxon rank sum *P* <.001

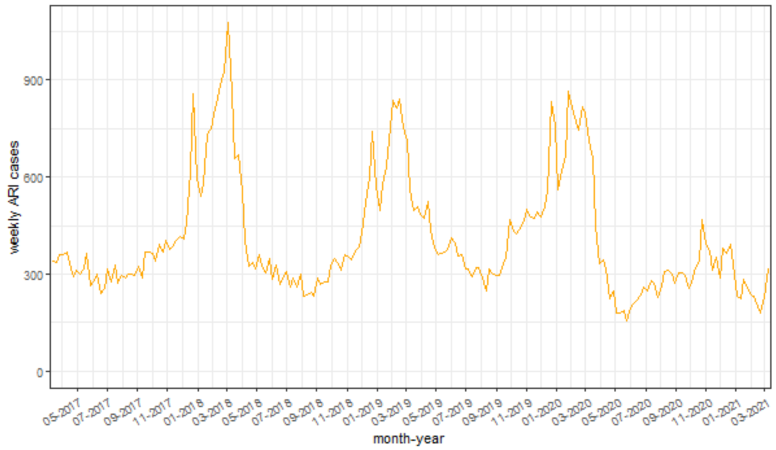

Supplemental Figure 3A

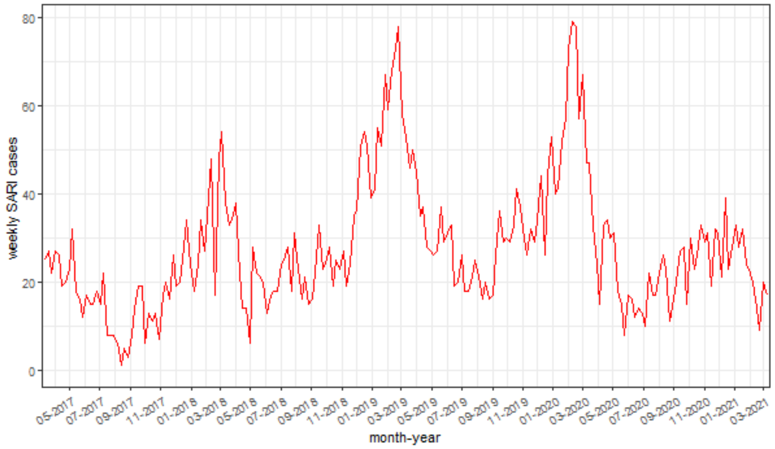

Supplemental Figure 3B

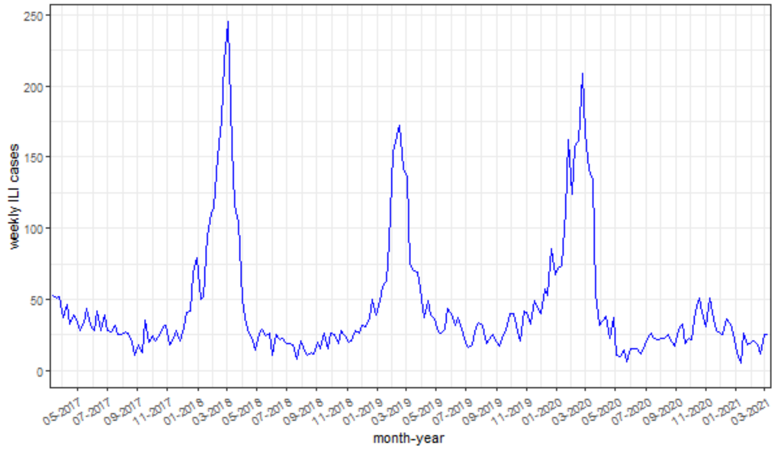

Supplemental Figure 3C

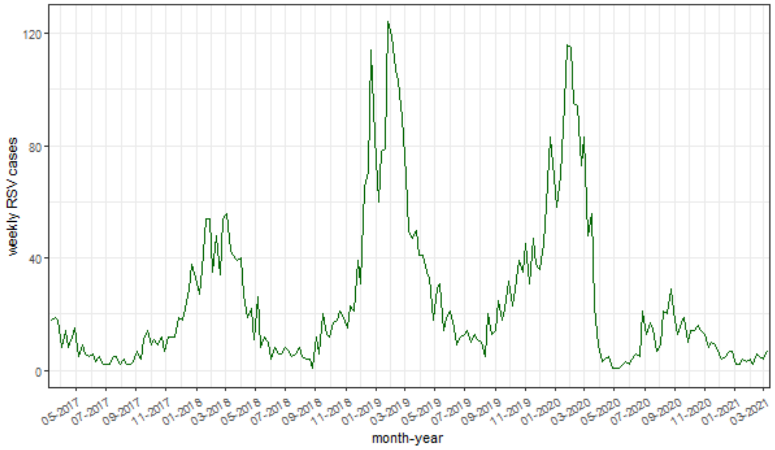

Supplemental Figure 3D

Supplemental Figure 3. Weekly case count of acute respiratory infection (ARI; 3A), severe acute respiratory infection (SARI; 3B), influenza-like-illness (ILI, 3C), and respiratory syncytial virus disease (RSV, 3D), between 6 March 2017 and 13 March 2021.
Cases of ILI and RSV both include probable and/or confirmed cases. Only one case definition was respectively used for ARI and SARI.

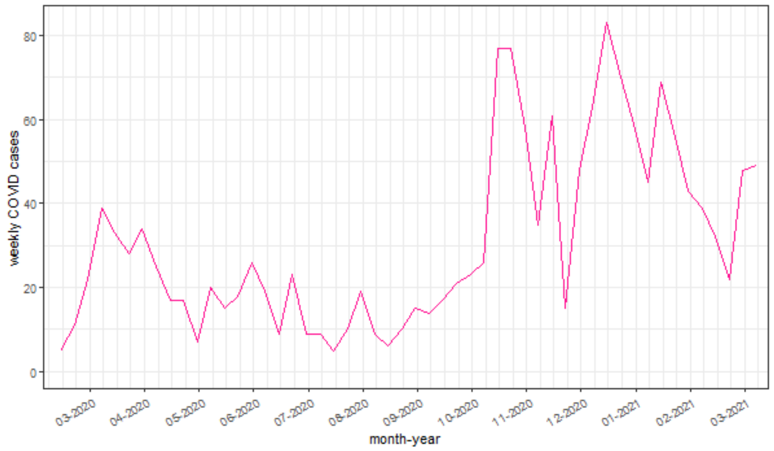

Supplemental Figure 4. Weekly case count of Coronavirus disease 2019 (COVID-19; probable + confirmed), between 6 March 2020 and including 13 March 2021.

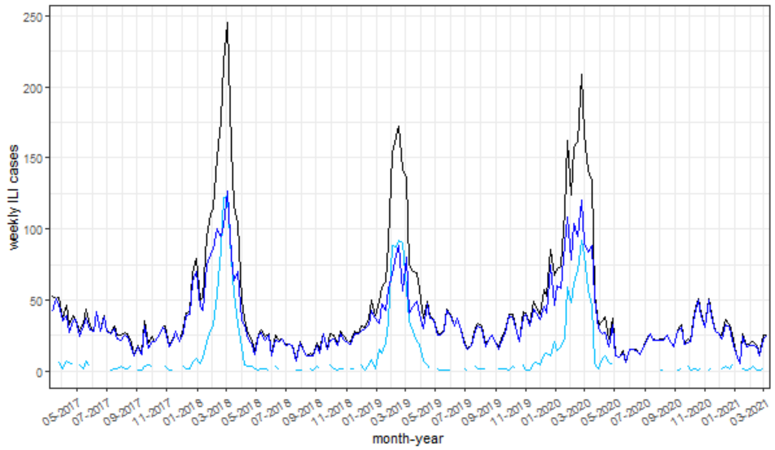

Supplemental Figure 5A: ILI cases (black), probable (dark blue), confirmed (light blue)

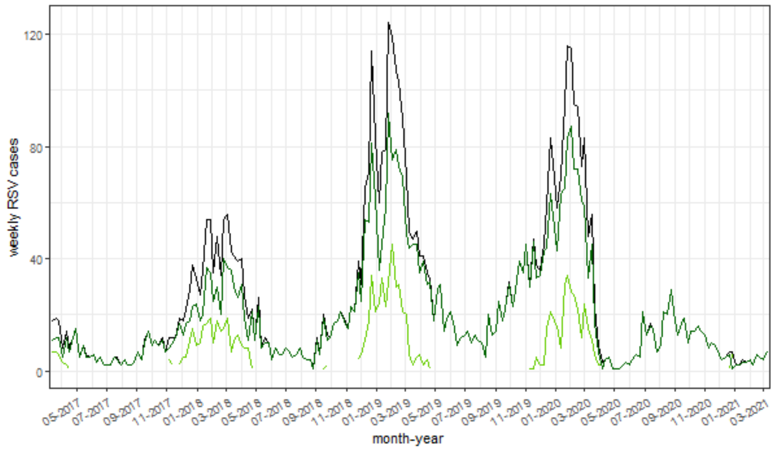

Supplemental Figure 5B: RSV cases (black), probable (dark green), confirmed (light green)

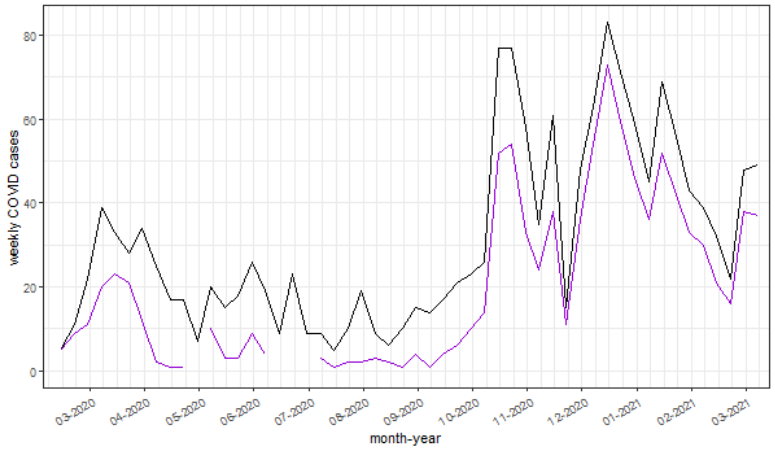

Supplemental Figure 5C: COVID-19 cases (black), confirmed (purple)

The combined case definition of COVID-19 cases equals the probable case definition.

Supplemental Figure 5. Weekly case counts, by probable and possible case classifications as well as the combined case definition, of influenza-like-illness (ILI, 5A), respiratory syncytial virus disease (RSV, 5B), and Coronavirus disease 2019 (COVID-19, 5C), between 6 March 2017 and 13 March 2021.
